# Studies on fatty acids and microbiota characterization of the gastrointestinal tract of Tianzhu white yaks

**DOI:** 10.3389/fmicb.2024.1508468

**Published:** 2025-01-17

**Authors:** Chen Shaopeng, Cui Changze, Qi Youpeng, Mi Baohong, Zhang Meixian, Jiao Chenyue, Zhu Chune, Wang Xiangyan, Hu Jiang, Shi Bingang, Ma Xueming, Zhao Zhidong, Zhang Xiaolan

**Affiliations:** ^1^Gansu Key Laboratory of Herbivorous Animal Biotechnology, College of Animal Science and Technology, Gansu Agricultural University, Lanzhou, China; ^2^Linxia Beef Cattle Industry Development Research Institute, Linxia, China; ^3^Livestock Industry Development Center of Hezheng County, Hezheng, Gansu, China

**Keywords:** Tianzhu white yak, volatile fatty acids, microbiota, gastrointestinal, function

## Abstract

**Introduction:**

The gut microbiota significantly influences the host’s production performance and health status, with different gastrointestinal tissues exhibiting functional diversity reflected in their microbial diversity.

**Methods:**

In this study, five adult male Tianzhu white yaks (4.5 years old) were selected and fed under the same nutritional conditions. After the feeding experiment, the yaks were slaughtered, and chyme samples were collected from the rumen, abomasum, jejunum, and colon for 16S rRNA full-length sequencing and volatile fatty acid analysis.

**Results:**

The results showed that the microbial composition and diversity of the rumen and abomasum were similar, with close genetic distances and functional projections. In contrast, the jejunum and colon had distinct microbial compositions and diversity compared to the rumen and abomasum. At the phylum level, the dominant phyla in the rumen, abomasum, and colon were Firmicutes and Bacteroidetes, while in the jejunum, the dominant phyla were Firmicutes and Proteobacteria. The abundance of Firmicutes differed significantly between the jejunum (87.24%) and the rumen (54.67%), abomasum (67.70%), and colon (65.77%). Similarly, Bacteroidetes showed significant differences between the jejunum (2.21%) and the rumen (36.54%), abomasum (23.81%), and colon (28.12%). At the genus level, *Rikenellaceae_RC9_gut_group* and *Christensenellaceae_R-7_group* were dominant in both the rumen and abomasum. In the jejunum, Romboutsia and *Paeniclostridium* were dominant, while *Rikenellaceae_RC9_gut_group* and *UCG-005* were the dominant genera in the colon. At the species level, *rumen_bacterium_g_Rikenellaceae_RC9_gut_group* and *rumen_bacterium_g_Christensenellaceae_R-7_group* were dominant in both the rumen and abomasum, while *Clostridium_sp._g_Romboutsia* and *bacterium_g_Paeniclostridium* were unique to the jejunum. *Ruminococcaceae_bacterium_g_UCG-005* and *bacterium_g_Rikenellaceae_RC9_gut_group* were unique to the colon. KEGG functional prediction of the microbiota indicated that the dominant functions in the rumen, abomasum, colon, and jejunum were amino acid metabolism, glycan biosynthesis and metabolism, carbohydrate metabolism, and membrane transport, respectively, reflecting the digestive functions of these organs. Volatile fatty acid analysis showed that the concentrations of acetic acid, propionic acid, and butyric acid in the rumen were significantly higher than those in the abomasum, jejunum, and colon (*p* < 0.05). Among these, the propionic acid concentration in the jejunum was significantly lower than in the abomasum and colon. Additionally, correlation analysis results indicated that acetic acid and butyric acid were significantly positively correlated with the ruminal bacterial community (*p* < 0.05). The total volatile fatty acid concentration was highest in the rumen, decreased to less than one-fifth of the rumen’s total volatile fatty acid concentration in the abomasum and jejunum, and then reached a second peak in the colon.

**Conclusion:**

This study explored the microbial composition and differential bacterial genera in the rumen and intestines of Tianzhu white yak, comparing the differences in volatile fatty acid levels and microbial composition and function across different regions. This is important for understanding their gastrointestinal microbiota’s spatial heterogeneity.

## 1 Introduction

The yak (*Bos grunniens*), an ancient domesticated species, is primarily distributed across the Qinghai–Tibet Plateau and its surrounding regions (Shah et al., 2023). Due to its exceptional cold resistance and adaptability to high-altitude environments, the yak has become a vital resource for the livelihood and production of local herders. The Tianzhu white yak, a prized breed, is renowned for its distinctive white fur, superior meat quality, and strong environmental adaptability. This breed is mainly found in the Tianzhu Tibetan Autonomous County of Gansu Province, serving as a cornerstone of the region’s economic development and livestock industry. Tianzhu white yak meat is rich in high-quality proteins, unsaturated fatty acids, B vitamins, and various trace elements. These nutrients play a crucial role in human health, particularly for those living in high-altitude areas, by significantly aiding in physical strength replenishment and immune enhancement. However, the nutritional status of yaks is severely constrained by environmental changes, including outdated grassland management, pasture degradation, and especially the scarcity of forage during the long winter and spring seasons, leading to significant weight loss in yaks during winter and low meat production. This severely restricts the economic benefits and sustainable development of the yak industry. Consequently, improving yak growth performance to enhance meat production has become a focal point of yak research.

The gastrointestinal tract (GIT) is a highly complex organ where multiple dynamic physiological processes are tightly coordinated and interact with a dense and highly diverse microbiota (Parker et al., 2018). From its establishment in early life to the host-microbiota symbiosis in adulthood, the gut microbiota plays a critical role in our development and health. Microbes are especially vital for ruminants, as they can ferment plant fibers, which most monogastric animals cannot digest, producing volatile fatty acids (VFAs) and microbial proteins that provide energy and nutrients to the host. This process is also crucial for humans, who cannot digest and utilize fibrous plant materials, as it converts energy from plant photosynthesis into consumable products such as milk and beef (Sommer and Bäckhed, 2013). Increasing evidence suggests that the complex and diverse GIT microbiome is a fundamental factor influencing ruminant productivity and the quality of related milk and meat products (Ben Shabat et al., 2016). Studies indicate that the composition, structure, and abundance of functional microbial species in the ruminant digestive tract are closely linked to host energy absorption and susceptibility to gastrointestinal diseases. In practical production, adjusting the composition and structure of the digestive tract microbiota can improve the host’s physiological functions (Amanullah et al., 2021; Dewanckele et al., 2020; Gargallo et al., 2020; Matthews et al., 2019; Cummings and Macfarlane, 1991; McNeil, 2018). Therefore, it is essential to investigate the structure of its gastrointestinal microbiota and its relationship with VFAs. Currently, many studies have used 16S rRNA high-throughput sequencing to investigate the rumen and fecal microbiota of yaks, but research on other regions of the GIT is still lacking. This study analyzes the composition and differential bacterial genera in the rumen and intestines of Tianzhu white yaks, comparing the microbial composition and functional differences in different sections to provide foundational data for understanding the gut microbiota of yaks.

## 2 Materials and methods

### 2.1 Animals and sample collection

All animal experiments, including experimental design and feeding management, were approved by the Animal Ethics Committee of Gansu Agricultural University (Approval No. GSAU-Eth-AST-2023-034). The experiment was conducted in Heimaquanhai Village, Tianzhu County, Gansu Province (altitude: 2800 m). Healthy adult male yaks (4.5 years old, *n* = 5) with good body condition, raised in the same barn, were selected for the study. The experimental diet was formulated based on the “Feeding Standard of Beef Cattle” (NY/T 815–2004) with a high nutritional level of compound feed consisting of roughage and concentrate. The roughage included corn stalks, oat hay, and Astragalus stalks, while the concentrate comprised corn, bran, rapeseed meal, soybean protein powder, salt, and premix. The nutritional ratio of the diet was set at 65: 35, with a net energy (NEmf) of 97.84 MJ/kg. The composition and nutritional content of the diet are detailed in Supplementary Table S1. The compound feed was produced by Xinuo Agriculture and Animal Husbandry Co., Ltd., Minle County, Gansu Province. The animals were slaughtered at 4.5 years of age, and immediately after slaughter, the four segments of the gastrointestinal tract from each yak, including two segments of the stomach (rumen and abomasum) and two segments of the intestines (jejunum and colon), were dissected from the mesentery using a scalpel. Each segment was placed on a sterile plate, and the gastrointestinal contents were sampled into 5 mL cryotubes using sterile medical gloves. All samples were rapidly frozen in liquid nitrogen and stored at −80°C after being transported back to the laboratory.

### 2.2 Determination of gastrointestinal VFAs

For the extraction of gastrointestinal VFAs, the contents of the rumen, abomasum, jejunum, and colon, stored at −80°C, were thawed. A 5-mL sample of each was taken and placed into centrifuge tubes and centrifuged at 5400 rpm for 10 min. The supernatant was transferred into 1.5 mL centrifuge tubes, and 0.2 mL of perchloric acid solution containing 25% internal standard 2 EB was added. The mixture was incubated in an ice-water bath for 50 min and then centrifuged at 10,000 rpm for 10 min. The supernatant was filtered through a 0.22-μm membrane into brown vials for determination. The concentration of VFAs in the GIT was determined using a gas chromatograph (Agilent 6890N). The chromatographic column used was an HP1909IN-213 capillary column, and a micro-syringe was used to inject 1 μL of the sample into the injection port for determination. The chromatographic conditions were as follows: injection port temperature set at 220°C; program temperature mode set at 120°C for 3 min, then increased to 180°C for 1 min; N_2_ flow rate maintained at 2.0 mL/min.

### 2.3 16S rRNA gene sequencing

DNA was extracted using the E.Z.N.A. Soil DNA Kit (Omega Bio-tek, Norcross, GA, USA) according to the manufacturer’s instructions, and the concentration and purity of the DNA were checked using a NanoDrop2000 (Thermo Scientific Inc., Waltham, MA, USA). The integrity of the DNA was verified by 1% agarose gel electrophoresis. The DNA was then used as a template for PCR amplification of the 16S rRNA gene, with forward primer 27F (5′-AGAGTTTGATCCTGGCTCAG-3′) and reverse primer 1492R (5′-TACGGCTACCTTGTTACGACTT-3′). The PCR instrument was an ABI GeneAmp 9700 (ABI, Los Angeles, CA, USA). The reaction system was 4 μL of 5× TransStart FastPfu buffer, two μL of 2.5 mM dNTPs, 0.8 μL of forward primer (5 μM), 0.8 μL of reverse primer (5 μM), 0.4 μL of TransStart FastPfu DNA polymerase, 0.2 μL of BSA, and 10 ng of template DNA, for a total volume of 20 μL. All samples were amplified in triplicate. The PCR products were extracted from 2% agarose gels and purified using the AxyPrep DNA Gel Extraction Kit (Axygen Biosciences, Union City, CA, USA) according to the manufacturer’s instructions and then quantified using a Quantus™ Fluorometer (Promega, Madison, WI, USA). The purified PCR products were used to construct libraries using the NEXTFLEX Rapid DNA-Seq Kit (PerkinElmer, Waltham, MA, USA) and sequenced on the MiSeq PE300 platform.

### 2.4 Statistical analysis

After quality control and merging, the DADA2 plugin in the Qiime2 software was used to denoise the optimized sequences. Chloroplast and mitochondrial sequences annotated in all samples were removed. To minimize the impact of sequencing depth on subsequent alpha and beta diversity data analyses, the sequence count for all samples was normalized to 20,000. After normalization, the average sequence coverage for each sample (Good’s coverage) remained at 99.09%. Taxonomic analysis of OTUs was conducted using the Naive Bayes classifier in Qiime2 based on the SILVA 16S rRNA gene database (v 138). Functional prediction analysis of the 16S sequences was performed using PICRUSt2 (version 2.2.0). The following analyses, namely, Venn diagram analysis, rank-abundance curve, and pan/core species analysis, were conducted on all sequencing data and were performed using R language tools. Alpha diversity indices, including Chao 1, Shannon, Simpson, and ACE indices, were calculated using Mothur software, and intergroup differences in alpha diversity were analyzed using the Wilcoxon rank-sum test. Principal coordinate analysis (PCoA) based on the Bray–Curtis distance algorithm was used to examine the similarity of microbial community structures between samples, with analysis of similarity (ANOSIM) used for intergroup and intragroup difference statistical analysis. Community bar plots were generated using R language tools. Linear discriminant analysis effect size (LEfSe) analysis (LDA > 4), (*p* < 0.05) was employed to identify bacterial groups with significant differences in abundance between groups from phylum to genus levels. Functional pathway abundance tables derived from PICRUSt2 were used for functional difference analysis between groups using STAMP software. Species with Spearman’s correlation |r| > 0.6 and *p-value of* < 0.05 were selected for correlation network analysis.

## 3 Results

### 3.1 Analysis of VFAs in the gastrointestinal tract of yaks

**Figure 1:**
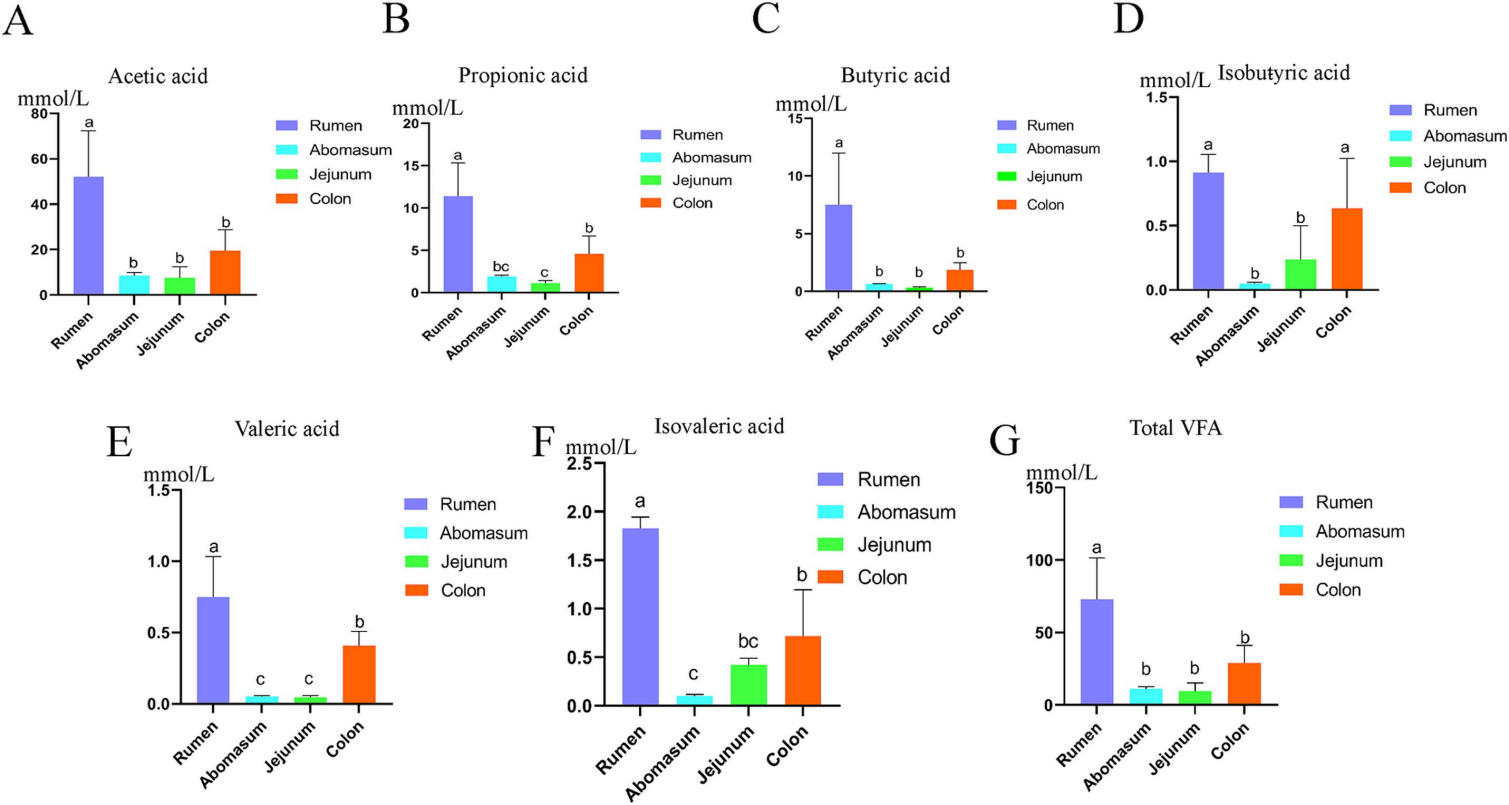
Fatty acid analysis of the gastrointestinal tract of yak. **(A–G)** Show the difference between acetic acid, propionic acid, butyric acid, isobutyric acid, valeric acid, and isovaleric acid and total acid contents among gastrointestinal groups using the single-factor ANOVA. In the figure, different letters a, b, and c represent significant differences (*p* < 0.05), while the same letters or those containing the same letters are not significant (*p* < 0.05).

We analyzed the differences in the contents of acetic acid, propionic acid, butyric acid, isobutyric acid, valeric acid, isovaleric acid, and total volatile fatty acids among the gastrointestinal groups using a one-way analysis of variance (ANOVA). The results showed (Figures 1A–G) that there are differences in volatile fatty acids in the contents of various gastrointestinal sites. The total volatile fatty acid content in the rumen was significantly higher than that in the abomasum, jejunum, and colon (*p* < 0.05), with acetic, propionic, and butyric acids also being significantly higher in the rumen compared to the abomasum, jejunum, and colon (*p* < 0.05). The isobutyric acid content in the rumen and colon was significantly higher than that in the abomasum and jejunum (*p* < 0.05); the valeric acid content in the rumen was significantly higher than that in the colon, while the valeric acid content in the abomasum and jejunum was significantly lower than that in the rumen and colon (*p* < 0.05). The isovaleric acid content showed that the rumen was significantly higher than the colon, jejunum, and abomasum, and the colon was significantly higher than the abomasum (*p* < 0.05).

### 3.2 Sequencing results and microbial diversity

**Figure 2:**
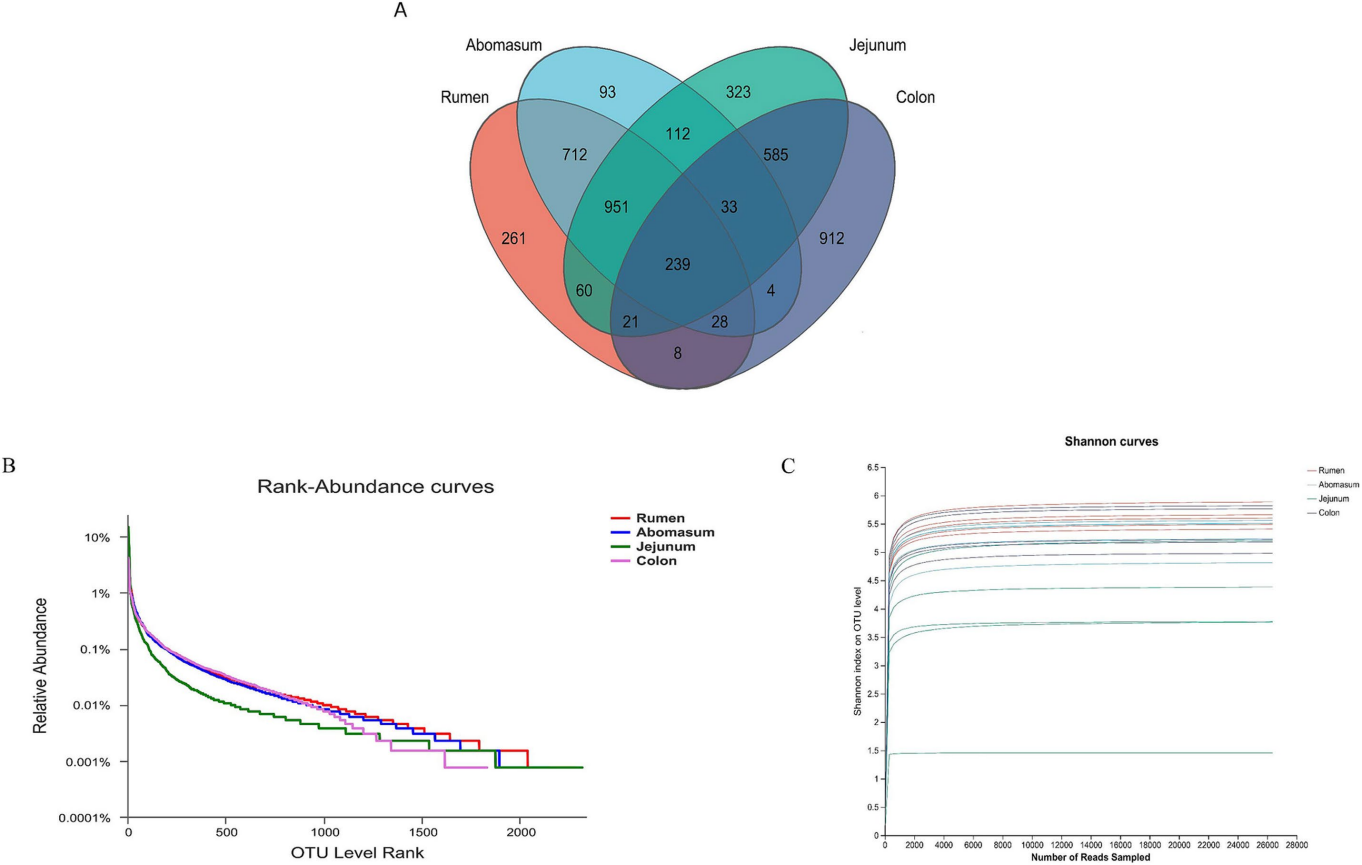
**(A)** Venn diagram analysis of species based on OTU level. **(B)** Rank-abundance curves can be used to explain two aspects of diversity, namely, species richness and community evenness. In the horizontal direction, species richness is reflected by the curve’s width. The larger the range of the curve on the horizontal axis, the higher the species richness. The shape of the curve (flatness) reflects the uniformity of the community in the sample, and the gentler the curve, the more evenly distributed the species. **(C)** Pan/Core species analysis is used to describe changes in total species and core species as the sample size increases and is widely used to determine whether the sample size is adequate.

The 16S rRNA sequencing of 20 samples from 5 cows yielded 1,235,974 optimized sequences after quality control, sequence orientation correction, and primer removal. Non-redundant sequences were clustered into operational taxonomic units (OTUs) at 97% similarity, resulting in 4,342 OTUs after removing chimeras. To understand the common and unique features of the microbiota in different gastrointestinal regions, a Venn diagram (Figure 2A) was used to examine shared OTUs among these regions. The rumen and abomasum shared 712 OTUs, while the jejunum had 323 unique OTUs and the colon had 912 unique OTUs. The results indicated that the microbial abundance was highest in the large intestine, followed by the stomach and the small intestine. The rank-abundance curve (Figure 2B) demonstrated a sequential decrease in microbial abundance among the four groups: jejunum, rumen, abomasum, and colon. The microbial diversity in the rumen and abomasum was higher than in the colon, with the lowest diversity in the jejunum, suggesting a high dominance of specific microbial populations in the jejunum samples.

To verify the accuracy of the analysis, *α*-diversity indices including Shannon, Simpson, Ace, and Chao1 indices were calculated (Table 1). The results showed that the Shannon index in the jejunum was significantly lower than in the rumen, abomasum, and colon (*p* < 0.05), while the Simpson index was significantly higher in the jejunum than in these three regions (*p* < 0.05). There were no significant differences in the Shannon and Simpson indices among the rumen, abomasum, and colon. The Ace index was significantly higher in the rumen than in the jejunum and colon, and the abomasum was significantly higher than the jejunum (*p* < 0.05). The Chao1 index was significantly higher in the rumen and abomasum than in the jejunum and colon (*p* < 0.05), with no significant differences between the rumen and abomasum or between the jejunum and colon. The Good’s coverage for each sample was above 90%, and the curve tended to flatten, indicating that the sequencing data sufficiently covered each bacterial community (Figure 2C).

**Table 1:** Analysis of microbial *α* diversity in different parts of the gastrointestinal tract of Tianzhu white yak.

Item	Shannon	Simpson	Ace	Chao1
Rumen	5.62 ± 0.18^a^	0.010 ± 0.003^b^	1547.97 ± 112.72^a^	1530.33 ± 114.28^a^
Abomasum	5.33 ± 0.30^a^	0.021 ± 0.020^b^	1449.49 ± 103.74^ab^	1147.16 ± 106.53^ab^
Jejunum	4.29 ± 0.70^b^	0.066 ± 0.034^a^	1016.04 ± 472.30^c^	1016.23 ± 470.39^c^
Colon	5.40 ± 0.37^a^	0.021 ± 0.015^b^	1205.81 ± 64.51^bc^	1179.11 ± 63.02b^c^

a, b, and c in the same column of the above table indicate significant differences (*p* < 0.05), while the same letters indicate no significant differences (*p* > 0.05).

### 3.3 Taxonomic composition of gastrointestinal microbiota

**Figure 3:**
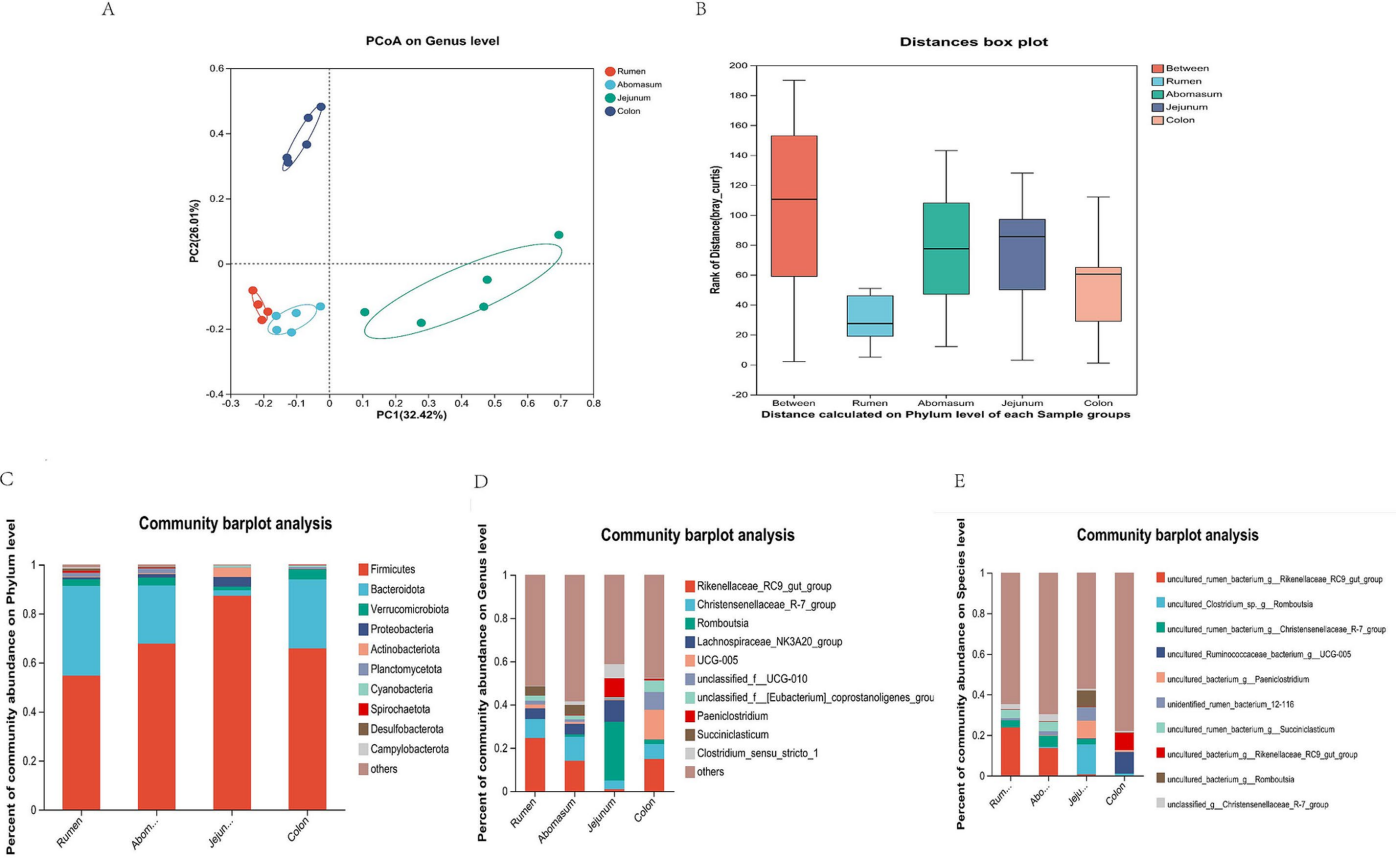
Composition and proportion of microorganisms. **(A)** Principal coordinate analysis (PCoA) based on all samples. **(B)** ANOSIM. **(C)** Phylum-level microbial composition at each site. **(D)** Generic microbial composition of each site. **(E)** Species-level microbial composition at each site.

We analyzed the microbial community composition in four gastrointestinal regions. We found that the rumen and abomasum had similar microbial compositions. At the same time, the rumen–abomasum cluster was separated from the jejunum and colon clusters, with principal component 1 (PC1) and principal component 2 (PC2) explaining 34.42 and 26.01% of the variation, respectively (Figure 3A). The results indicated differences in microbial community composition among different gastrointestinal regions, further supported by ANOSIM analysis, which showed that intergroup differences were more significant than intragroup differences (Figure 3B). In the taxonomic composition analysis, we focused on the top 10 bacterial taxa at the phylum, genus, and species levels, and the top three bacteria with the highest abundance in each group were selected as the dominant bacteria. At the phylum level, Firmicutes and Bacteroidota were the dominant phyla in the rumen, abomasum, and colon, while Firmicutes and Proteobacteria were dominant in the jejunum (Figure 3C). Significant differences were observed for Firmicutes between the jejunum (87.24%) and the rumen (54.67%), abomasum (67.70%), and colon (65.77%). Bacteroidota also showed significant differences between the jejunum (2.21%) and the rumen (36.54%), abomasum (23.81%), and colon (28.12%). At the genus level, *Rikenellaceae_RC9_gut_group* (rumen: 24.52%, abomasum: 13.98%), *Christensenellaceae_R-7_group* (rumen: 8.75%, abomasum: 11.10%), and *Lachnospiraceae_NK3A20_group* (rumen: 4.90%, abomasum: 4.82%) were the shared dominant genera in the rumen and abomasum. In the jejunum, *Romboutsia* (27.05%) and *Lachnospiraceae_NK3A20_group* (10.10%) were the dominant genera; *Rikenellaceae_RC9_gut_group* (14.81%), *UCG-005* (13.71%), and *unclassified_f_UCG-010* (8.24%) were dominant in the colon (Figure 3D). At the species level, *uncultured_rumen_bacterium_g_Rikenellaceae_RC9_gut_group* (rumen: 23.70%, abomasum: 13.61%), *ncultured_rumen_bacterium_g__Christensenellaceae_R-7_group* (rumen: 3.58%, abomasum: 5.43%), and *uncultured_rumen_bacterium_g_Succiniclasticum* (rumen: 4.31%, abomasum: 4.65%)were the shared dominant species in the rumen and abomasum; *uncultured_Clostridium_sp._g_Romboutsia* (14.78%), *uncultured_bacterium_g_Paeniclostridium* (8.68%), and *uncultured_bacterium_g_Romboutsia* (8.16%)were the dominant species unique to the jejunum; *uncultured_Ruminococcaceae_bacterium_g_UCG-005* (10.60%) and *uncultured_bacterium_g_Rikenellaceae_RC9_gut_group* (8.52%) were the dominant species unique to the colon (Figure 3E).

### 3.4 Biomarkers of the stomach, small intestine, and large intestine

**Figure 4:**
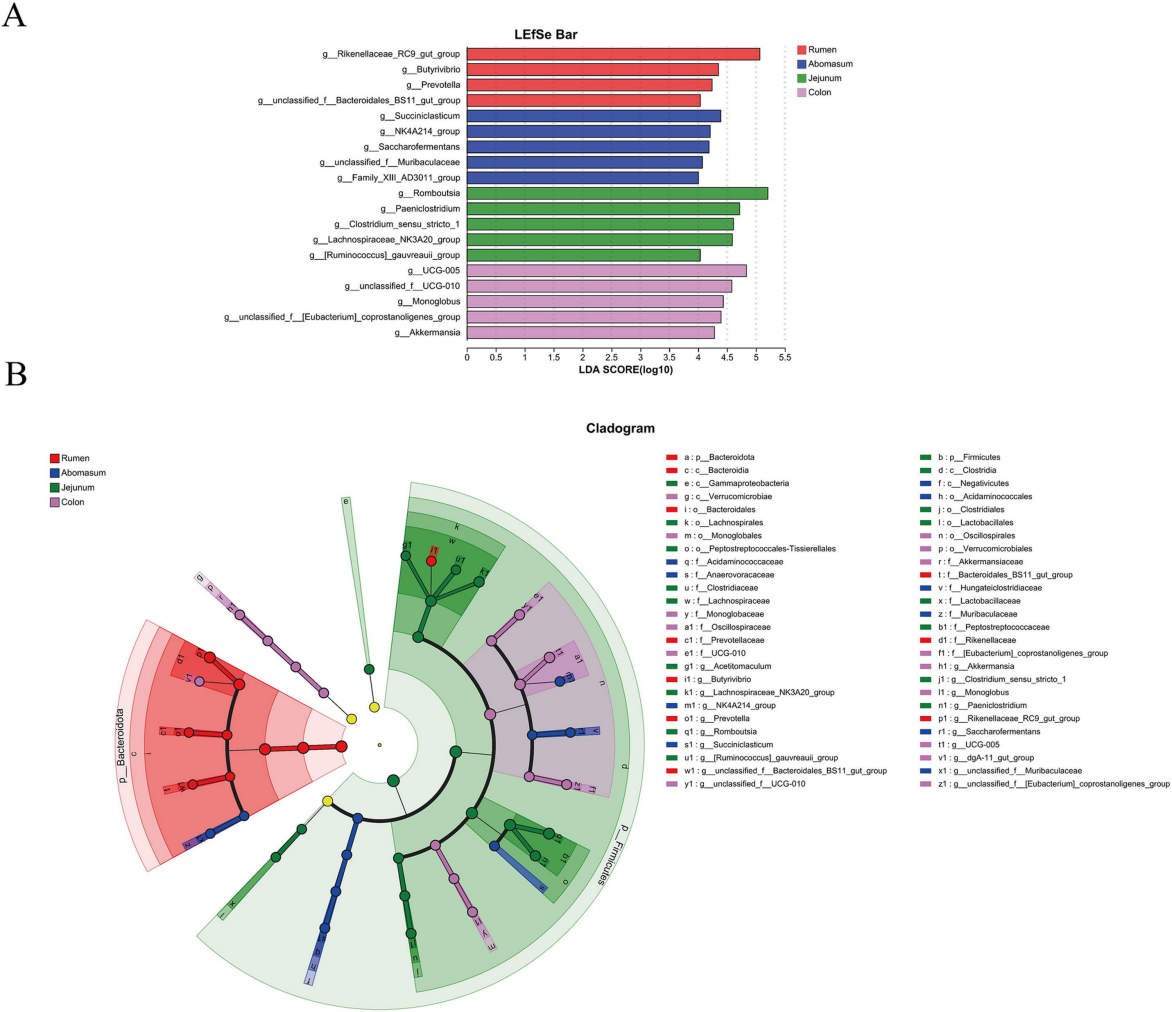
Linear discriminant analysis (LDA) effect size (LEfSe) analysis of gastrointestinal segments in Tianzhu white yak. LEfSe analysis histogram of each gastrointestinal segment **(A)**. The ordinate is the significantly different taxa between the groups, and the abscess is a bar chart showing the LDA logarithmic fraction value for each taxon. The longer the length, the more significant the difference in taxa. **(B)** LEfSe analysis of each segment of the gastrointestinal tract. Node size corresponds to the average relative abundance of taxa, and hollow nodes represent taxa that do not differ significantly between taxa. These letters identify taxa names that vary widely between groups.

We employed linear discriminant analysis effect size (LEfSe) for multi-level species difference analysis across groups to identify biomarkers for different gastrointestinal regions. Using LDA scores and *p*-values (LDA score > 4, *p* < 0.05), we compared the microbial abundance and differences among groups, screening differential species. The results (Figures 4A,B) showed that 10, 10, 17, and 15 biomarkers were identified in the rumen, abomasum, jejunum, and colon groups. The rumen group was mainly characterized by biomarkers such as *Rikenellaceae_RC9_gut_group*, *Prevotella*, *Butyrivibrio*, and *Bacteroidales_BS11_gut_group*.

In the abomasum, significantly enriched bacteria included *Succiniclasticum*, *NK4A214_group*, *Saccharofermentans*, *Muribaculaceae*, and *Family_XIII_AD3011_group*. The jejunum was mainly characterized by differential microbes such as *Romboutsia*, *Paeniclostridium*, *Lachnospiraceae_NK3A20_group*, *[Ruminococcus]_gauvreauii_group*, and *Clostridium_sensu_stricto_1*. *UCG-005*, *[Eubacterium]_coprostanoligenes_group*, *UCG-010*, *Monoglobus*, and *Akkermansia* were identified as significant differential microbes in the colon.

### 3.5 KEGG functional prediction

**Figure 5:**
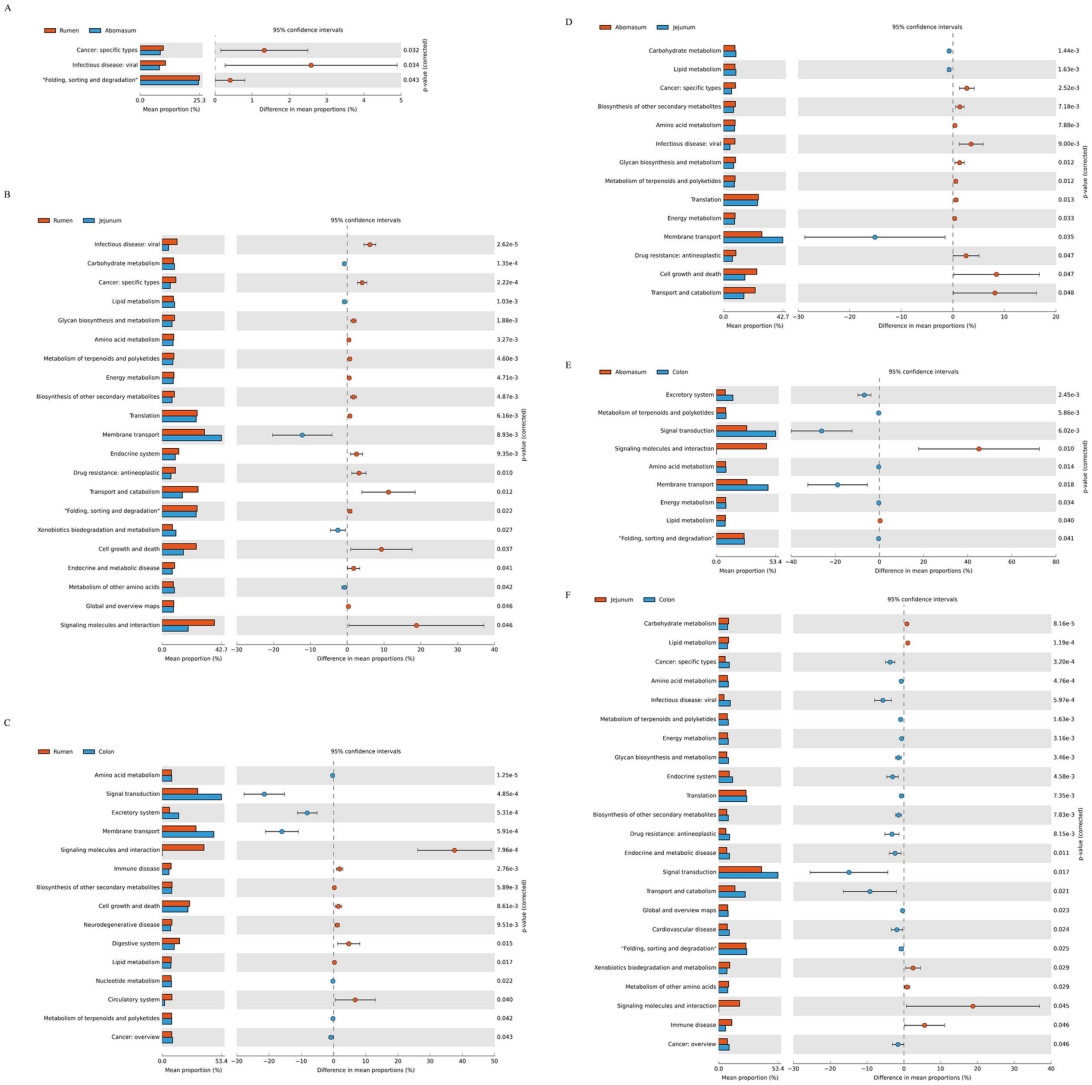
Enrichment analysis of KEGG metabolic pathways. **(A–F)** Show the differential analyses of metabolic pathways between the following pairs: rumen and abomasum, rumen and jejunum, rumen and colon, abomasum and jejunum, and jejunum and colon, respectively.

Using the Kyoto Encyclopedia of Genes and Genomes (KEGG) pathway prediction (Figure 5), we analyzed functional differences in gastrointestinal bacteria among the four groups. Significant functional differences were observed in bacteria from different gastrointestinal regions at the secondary level of KEGG metabolic pathways. Between the rumen and abomasum groups (Figure 5A), protein folding, sorting, and degradation were more active in the rumen (*p* < 0.05). Between the rumen and jejunum (Figure 5B), membrane transport, xenobiotic degradation and metabolism, and other amino acid metabolism were more active in the jejunum (*p* < 0.05), whereas the rumen was mainly enriched in transport and catabolism, cell growth and death, and signal transduction pathways. Between the rumen and colon (Figure 5C), signal transduction, excretory system, and membrane transport were more abundant in the colon, while the rumen was enriched in pathways related to immune diseases, synthesis of other secondary metabolites, lipid metabolism, and the circulatory system. Between the abomasum and jejunum (Figure 5D) and abomasum and colon (Figure 5E), biosynthesis of other secondary metabolites, amino acid metabolism, glycan biosynthesis and metabolism, terpene and polyketide metabolism, translation, and energy metabolism were mainly enriched in the abomasum. In contrast, carbohydrate metabolism, lipid metabolism, and membrane transport were mainly enriched in the jejunum.

### 3.6 Correlation analysis of gastrointestinal fermentation parameters and microbial communities

**Figure 6:**
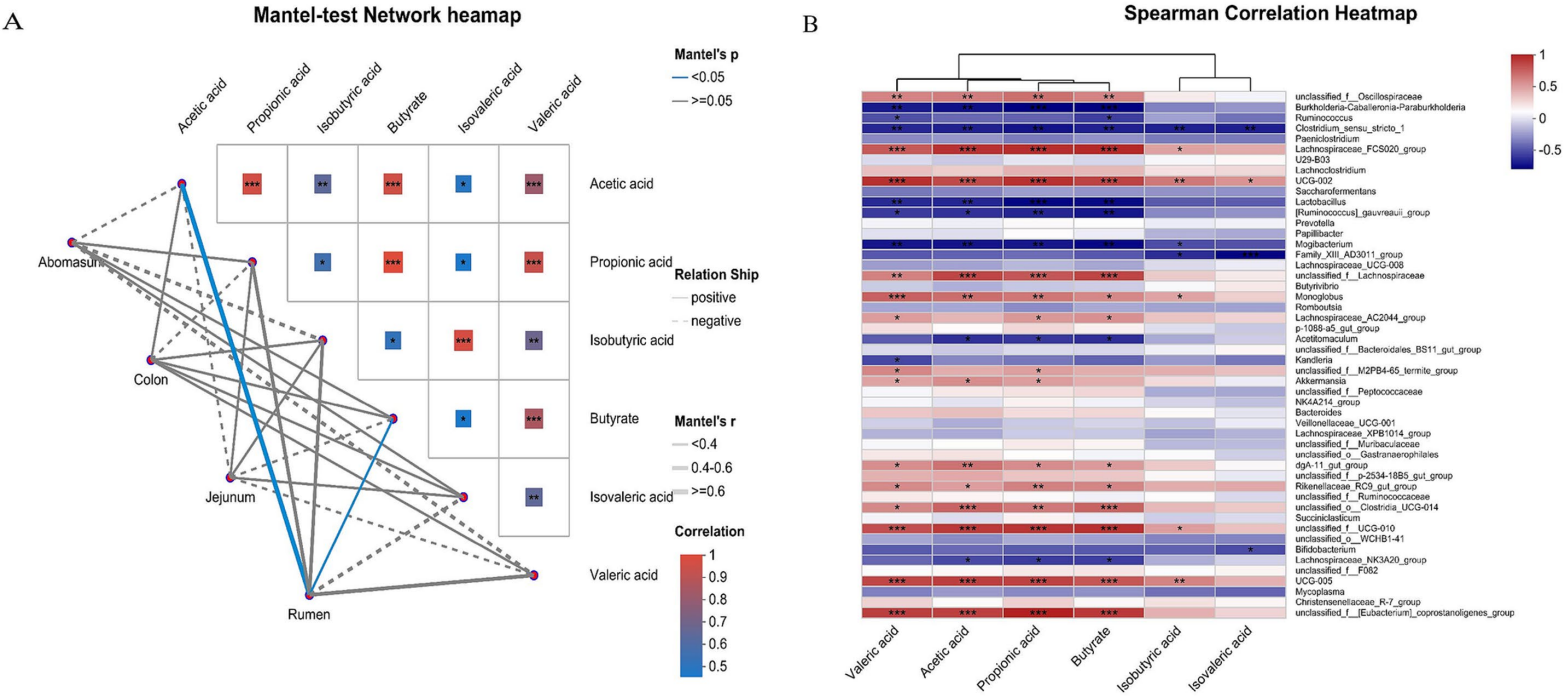
Correlation analysis of gastrointestinal microorganisms and VFAs in yaks. The Mantel-test network heatmap **(A)** shows the correlation between gastrointestinal fermentation parameters and microbial community structure. The lines in the figure represent the correlation between community and environmental factors, and the heatmap represents the correlation between environmental factors. Line thickness indicates the correlation between community and environmental factors is drawn with Mantel’r (absolute value of *r*). The relationship is categorized as Positive and Negative, indicating positive and negative correlations between community and environmental factors. Different colors in the heatmap represent positive and negative correlations, color depth represents the magnitude of positive and negative correlations, and asterisks in color blocks represent significance, *0.01 < *p* ≤ 0.05, **0.001 < *p* ≤ 0.01, and ***p ≤ 0.001. Spearman’s correlation heatmap **(B)** shows the correlation between species and environmental factors (X and Y axes are environmental factors and species, respectively, and R-and *p*-values were obtained by calculation. The R values are shown in different colors in the chart. If the *p*-value is less than 0.05, it is marked with a *. The legend on the right is the color interval of the different R values. **0.001, *0.01 < 0.05, or less *p* < *p* 0.01, or less ***p < 0.001 or less).

Using the Mantel test, we analyzed the correlation between fermentation parameters and microbial community structure (Figure 6A). The results showed that acetic acids and butyric acids were significantly positively correlated with the bacterial communities in the rumen group (*p* < 0.05). Spearman’s correlation analysis between fermentation parameters and gastrointestinal microbiota (Figure 6B) revealed that *Lachnospiraceae_FCS020_group, UCG-002*, *UCG-005, unclassified_f_[Eubacterium]_coprostanoligenes_group*, *unclassified_f_Lachnospiraceae and unclassified_f_UCG-010* were significantly positively correlated with acetic acids, propionic acids, butyric acids, and valeric acid (*p* < 0.001). *Lactobacillus, Burkholderia-Caballeronia-Paraburkholderia, Clostridium_sensu_stricto_1, Mogibacterium, and [Ruminococcus]_gauvreauii_group* were significantly negatively correlated with acids, butyric acids, and valeric acid (*p* < 0.01), and *Clostridium_sensu_stricto_1 and Family_XIII_AD3011_group* were significantly negatively correlated with isobutyric acid and isovaleric acid (*p* < 0.05).

## 4 Discussion

To maintain the regular energy supply, nutrient absorption, and microbial ecological balance in the gastrointestinal tract of Tianzhu white yak, significant differences in volatile fatty acid (VFA) levels were observed across different sections of the tract. VFAs such as acetic acid, propionic acid, and butyric acid are produced by the fermentation of carbohydrates and proteins by gastrointestinal microorganisms and play a critical role in physiological functions (McNeil, 2018; Cummings and Macfarlane, 1991). The majority of the VFAs generated in the rumen are absorbed in the rumen, reticulum, and omasum, with only a small amount entering the abomasum. The absorbed VFAs provide approximately two-thirds of the total energy derived from absorbed nutrients. Among these, acetic acid serves as the primary precursor for fat synthesis; 2–5% of propionic acid is converted into lactate during absorption by rumen epithelial cells, while the remainder enters the portal vein and is either oxidized or converted into glucose in the liver. Butyric acid, during absorption by rumen epithelial cells, is primarily transformed into ketone bodies (*β*-hydroxybutyrate, acetoacetate, and acetone), with β-hydroxybutyrate being a significant energy source for rumen epithelium. VFAs stimulate the growth and proliferation of intestinal epithelial cells, enhancing absorption capacity and improving nutrient utilization efficiency from feed by promoting nutrient transport and absorption (Dai et al., 2022). The VFAs produced by the fermentation of carbohydrates in feed by rumen microorganisms are primarily composed of acetic acid, propionic acid, and butyric acid in a ratio of approximately 70:20:10 (Faichney, 1968, 1969; Glinsky et al., 1976; Hoover and Clarke, 2023; Johnson and McBee, 2023; Stevens, 1978; Yang et al., 2023). Furthermore, VFAs support gut health by influencing microbial metabolism and growth, promoting the proliferation of beneficial microbes, and regulating the composition of gastrointestinal microbial communities. Studies on VFA concentrations in different sections of the gastrointestinal tract of various ruminants have shown that VFA concentrations are highest in the rumen, drop to less than one-fifth of rumen concentrations in the small intestine, and exhibit a secondary peak in the cecum and colon (Elsden et al., 1946). In this study, the concentrations of individual VFAs (acetic acid, propionic acid, butyric acid, isobutyric acid, valeric acid, and isovaleric acid) and total volatile fatty acids showed significant variation across groups. The general trend observed was that the highest concentrations were in the rumen, followed by a marked decrease in the abomasum and jejunum, and a pronounced peak in the colon. This could be attributed to differences in the degree of feed fermentation by microorganisms and the rate of VFA absorption in different gastrointestinal tract sections. The rumen, a sizeable anaerobic fermentation chamber, produces substantial amounts of VFAs, most of which are absorbed through the rumen epithelium, with the remainder absorbed in the abomasum, omasum, and other sections. This could explain the significant reduction in VFA levels from the rumen to the abomasum. In addition, undigested starch and glucose in the small intestine are fermented by bacteria to produce small amounts of VFAs. When the digestive processes in the reticulorumen are restricted, factors accelerating feed particle passage through the rumen reduce starch and fiber digestibility in the rumen, shifting part of the digestion to the proximal cecum and colon. In this study, microorganisms ferment the undigested feed to produce VFAs, which could explain the observed increase in VFA levels from the jejunum to the colon in this study. Interestingly, this study found a significant positive correlation between specific bacterial populations and the production of acetic acid and butyric acid in the rumen and numerous bacterial species associated with VFA levels in other sections of the gastrointestinal tract. These findings indicate that these bacteria are crucial in influencing gastrointestinal VFA levels. This suggests the potential to modify the gastrointestinal VFA profile of Tianzhu white yak by regulating bacterial community structure, thereby promoting host digestion and absorption.

The analysis of bacterial communities in different parts of the gastrointestinal tract of the Tianzhu white yak reveals variations in their composition and structure. Our study shows that the rumen, abomasum, and colon exhibit higher microbial diversity and sequentially decreasing microbial richness compared to the jejunum. Furthermore, our PCoA observed differences in microbial community composition between the rumen, abomasum, jejunum, and colon. There is a certain degree of similarity between the rumen and abomasum samples, whereas the samples representing the stomach, small intestine, and large intestine show distinctly different microbial community compositions. This indicates that the variation in microbial communities along different parts of the gastrointestinal tract confirms the spatial heterogeneity of the microbiota along the GI tract. These findings are consistent with some previous studies (de Oliveira et al., 2013; Mao et al., 2015; Wang et al., 2017), which may be due to the varying environmental factors in different parts of the gastrointestinal tract from the rumen to the small intestine and large intestine, such as temperature, humidity, pH, oxygen content, nutrient types, and digestion rates, leading to differences in microbial community structure (Longhi et al., 2020). In this study, Firmicutes dominate the entire gastrointestinal tract, accounting for 87.24% of the jejunum. Firmicutes are mainly responsible for producing volatile fatty acids by degrading starch, cellulose, hemicellulose, and oligosaccharides, while Bacteroidetes primarily function to degrade carbohydrates, proteins, and fats to generate energy (Flint et al., 2012). In addition, the ratio of Firmicutes to Bacteroidetes is closely related to fat deposition (Ley et al., 2006) and is influenced by the proportion of roughage (Patel et al., 2014). The study identifies *Christensenellaceae_R-7_group, Lachnospiraceae_NK3A20_group*, *Romboutsia, UCG-005*, and *unclassified_f__UCG-010* as predominant genera in the gastrointestinal tract. The *Christensenellaceae_R-7_group* is positively correlated with protein metabolism in feed and intestinal metabolites and can promote rumen development and increase the digestion and absorption of nutrients (Ma et al., 2022). The *Lachnospiraceae_NK3A20_group* is involved in the metabolism of various carbohydrates, especially pectin in feed, and is closely related to butyric acid production (Seshadri et al., 2018). *UCG-005* and *unclassified_f__UCG-010* are associated with starch and fiber degradation in ruminants, and these bacteria may contribute to further fermentation of feed in the colon (Gao et al., 2023; Ma et al., 2022). These results indicate that the genera above may be essential components of the gastrointestinal tract of the Tianzhu white yak and stably participate in the host’s growth.

The LEfSe analysis identified *Rikenellaceae_RC9_gut_group, Butyrivibrio*, and *Prevotella* as biomarkers of the rumen. *Rikenellaceae_RC9_gut_group*, a Gram-negative bacterium capable of degrading mucins, is a dominant genus in the gastrointestinal tract of ruminants (Gao et al., 2023). The *Rikenellaceae_RC9_gut_group* belongs to the Rikenellaceae family and represents a common group of anaerobic bacteria in the rumen, capable of participating in the degradation of cellulose and other plant polysaccharides. This is crucial for the rumen fermentation process in ruminants as the rumen is the primary site for digesting plant-based feed. The degradation of cellulose helps release energy and nutrients for the animal’s utilization. Studies have shown that *Rikenellaceae_RC9_gut_group* can digest and absorb most unfermented polysaccharides, producing volatile fatty acids (VFAs) such as acetic and butyric acid (Pei et al., 2010). Compared to other sites, the higher abundance of *Rikenellaceae_RC9_gut_group* in this study may be related to the high digestive efficiency of rumen microbes in breaking down fibrous and polysaccharide materials. *Butyrivibrio* is an important degrader and utilizer of lignocellulose in plant materials. Studies have found that *Butyrivibrio* encodes a diverse array of carbohydrate-active enzymes and binding proteins, with 1,706 carbohydrate-binding modules identified, which are predicted to be involved in the depolymerization and transport of insoluble plant polysaccharides (Palevich et al., 2019). Research using the KEGG, eggNOG, and CAZy databases to annotate the functions of *Prevotella* bacteria genes in the rumen of Holstein cows revealed enrichment in functions related to carbohydrate, amino acid, pyruvate, insulin, and lipid metabolism as well as transport (Wu et al., 2021). Both the *Butyrivibrio* genus and *Prevotella* genus possess strong cellulolytic capabilities. Among them, *Butyrivibrio* is a major producer of butyric acid, which can lower the pH in the rumen, inhibit the growth of pathogenic microorganisms, and maintain the stability of the microbiota (Rubino et al., 2017). The *Prevotella* genus provides energy to the host by fermenting various carbon sources (such as acetic acid, propionic acid, and butyric acid) (Xiao et al., 2016). In addition, *Prevotella* can influence gas production through hydrogen production pathways in synergy with other microorganisms (Perea et al., 2017). These genera are predominant in the rumen, which is likely related to the rumen’s role as a critical organ for cellulose digestion in ruminants. The abomasum, the only gastric compartment in ruminants containing digestive glands, primarily breaks down nutrients in the feed through chemical processes, including the digestion of proteins and the initial breakdown of fats. In our study, three bacterial genera—*Succiniclasticum, NK4A214_group, and Saccharofermentans*—were identified as specific microbiota of the abomasum. *Succiniclasticum* converts succinate into propionic acids through fermentation, enhancing the host’s bioavailability of butyric acids (Van Gylswyk, 1995). The *NK4A214_group* is primarily involved in fiber and starch degradation and positively correlates with amino acid metabolites, suggesting that bacterial carbohydrate breakdown may drive changes in amino acid metabolic pathways (Yi et al., 2022). *Saccharofermentans*, belonging to the Bacteroidetes phylum, includes 116 genes encoding glycoside hydrolases involved in the degradation of hemicellulose, pectin, arabinogalactan, starch, fructans, and chitin (Tomazetto et al., 2018). The *Succiniclasticum* genus primarily converts succinate into acetic and propionic acids through fermentative pathways, which play a key role in fiber digestion. It is particularly crucial in the degradation of complex carbohydrates, helping to improve energy utilization efficiency (Wang et al., 2021). The *NK4A214_group* genus in the abomasum mainly ferments certain polysaccharides and cellulose, producing VFAs as energy products. *NK4A214_group* plays an important role in improving the digestibility of plant-based feed in ruminants, especially for hard-to-digest fibrous materials (Liu et al., 2019). The *Saccharofermentans* genus is primarily involved in fermentation, particularly the metabolism of sugars, producing lactate and other VFAs (Zhang et al., 2021). *Saccharofermentans* help ruminants convert complex sugars into absorbable VFAs in the abomasum by fermenting carbohydrates, providing an additional energy source. Furthermore, *Saccharofermentans* help enhance the digestion of plant-based feed in ruminants and, to some extent, regulate the balance of the intestinal microbiota. These three genera play important roles in the microbiota of the ruminant abomasum, each supporting the animal’s energy absorption and digestion process through different metabolic pathways. Their combined action may help improve the digestion efficiency of plant-based feed in ruminants, enhance energy and nutrient utilization, and potentially positively affect intestinal health and immune function. The *[Ruminococcus]_gauvreauii_group* plays an important role in releasing energy through the degradation of high-starch materials (Ze et al., 2012). Its presence in the jejunum is likely associated with the further digestion and absorption of feed. Furthermore, the *[Ruminococcus]_gauvreauii_group* may occupy a specific niche within the complex ecosystem of the jejunum, collaborating with other microbial groups to jointly maintain intestinal health. *Lachnospiraceae_NK3A20_group* is positively correlated with propionic acid levels in the hindgut of sheep (Ma et al., 2023). *Clostridium_sensu_stricto_1* is significantly associated with colonic acetic acids, total SCFAs in the colon, and some metabolites (Yang et al., 2024). *Paeniclostridium*, a pathogenic bacterium, is abundant in cows with mastitis and lambs with intestinal inflammation (Gryaznova et al., 2021). In our study, these three bacteria were identified as biomarkers of the jejunum. The study found that *UCG_005, Akkermansia, and Monoglobus* are identified as specific colon microbiota. Studies have shown that *UCG_005* positively correlates with linoleic and conjugated linoleic acids, indicating that *UCG_005* from the family Ruminococcaceae may be involved in lipid metabolism (Zhao et al., 2024). *Akkermansia* is a beneficial microbe associated with neurodevelopment, digestion, and intestinal absorption in animals (Qu et al., 2023). Research indicates that the *Monoglobus* *genome* has a highly specialized glycoside hydrolase repertoire for pectin degradation, unique among the human gut’s Firmicutes.

In this study, we inferred the abundance of functional homologs in the gastrointestinal microbiome of Tianzhu white yak based on the KEGG database and performed a differential analysis of functional pathways between groups. We found that many metabolism-related pathways were enriched in the stomach and jejunum. In the rumen, amino acid and lipid metabolism pathways were predominantly enriched. Previous studies have shown that pathways such as phenylalanine, tyrosine, and tryptophan biosynthesis and phenylalanine metabolism are abundant in the rumen fluid of yaks fed for 6 months, with phenylalanine hydroxylase catalyzing the production of tyrosine, making phenylalanine an essential metabolic pathway (Yang et al., 2018). Both pathways are related to phenylalanine, highlighting its significance in rumen microbial metabolic pathways (Xu et al., 2021), and both belong to amino acid metabolism, indicating that our findings are consistent with previous studies. The abomasum is characterized by pathways related to glycan biosynthesis, metabolism, and energy metabolism, suggesting that the abomasal bacterial community plays a crucial role in assisting the host with digestion and nutrient transformation. The bacterial community in the jejunum also plays an indispensable role in assisting the host with nutrient digestion and absorption (Wang et al., 2021), with numerous enriched metabolic pathways such as carbohydrate metabolism, lipid metabolism, and other amino acid metabolism.

## 5 Conclusion

**Figure 7:**
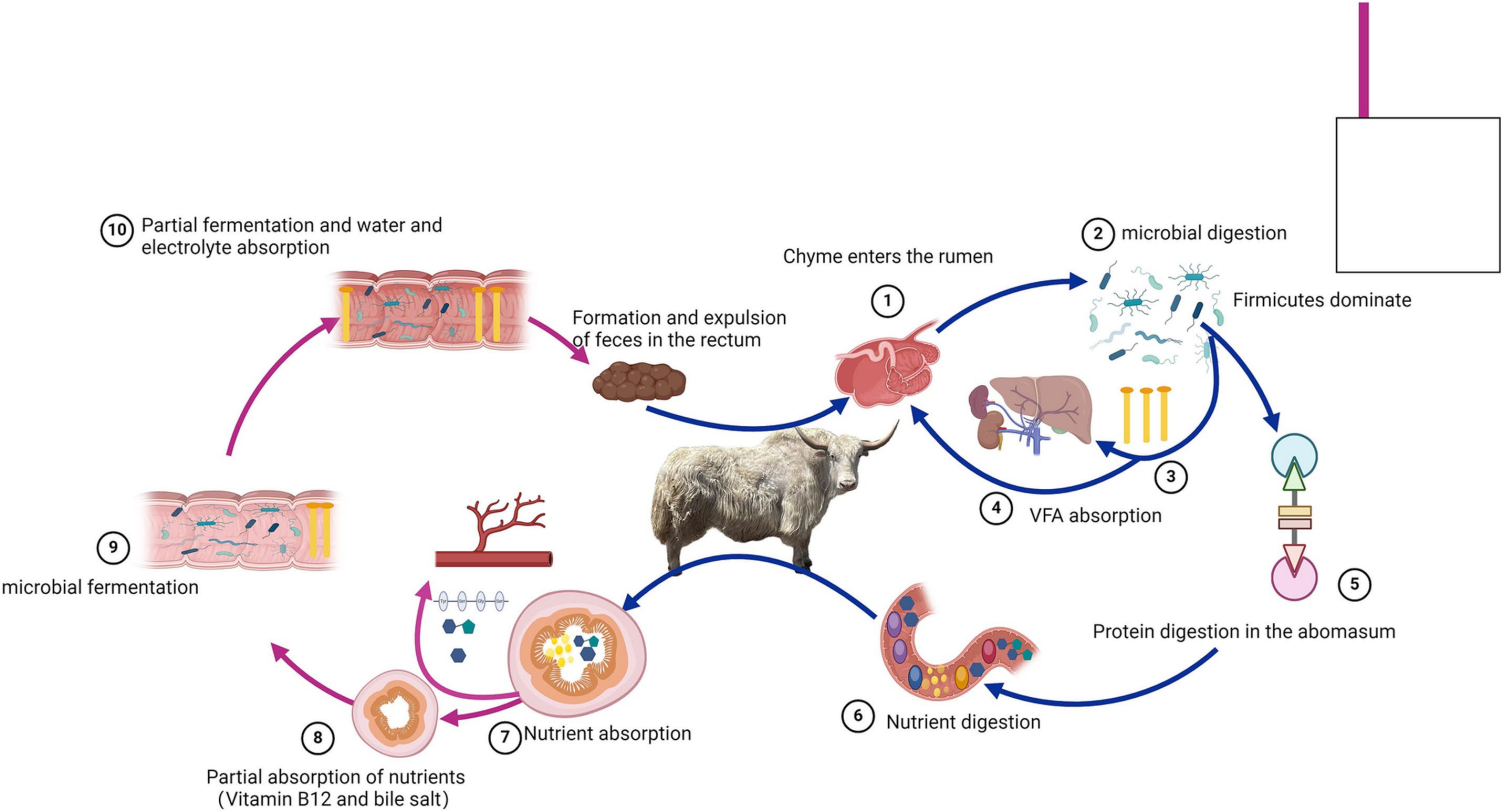
Overview of the gastrointestinal digestive process in Tianzhu white yak.

This study reveals the unique composition of the gastrointestinal microbiota and the trend of VFA concentrations in the gastrointestinal tract of the Tianzhu white yak, which thrives in alpine regions (Figure 7). The findings indicate significant differences in the bacterial composition and structure between different gastrointestinal tract sections, with each section exhibiting specific characteristics. In addition, the study highlights the solid metabolic functions of the rumen, abomasum, and jejunum. This research lays the foundation for understanding the complex gastrointestinal microbiome of the Tianzhu white yak. However, the study has limitations, such as a small sample size.

## Data Availability

The datasets presented in this study are available in online repositories. The name and accession number of the repository can be found in the article/Supplementary material. The Microbial sequencing data provided in this study can be found in the GenBank Sequence Read Archive (SRA) database under accession number PRJNA1165896.
