# Supplementary material for: Studies on fatty acids and microbiota characterization of the gastrointestinal tract of Tianzhu white yaks
Source: Front Microbiol. 2025 Jan 17;15:1508468. doi: 10.3389/fmicb.2024.1508468 (PMC11784337; doi:10.3389/fmicb.2024.1508468)
Supplement: Supplementary file 1 [file Table_1.DOCX]

| **项目Items** | **Content/%** |  |
| --- | --- | --- |
| 日粮组成 Diet composition |  |  |
| 玉米秸秆 Corn straw | 38.90 |  |
| 燕麦草 Oat grass | 13.30 |  |
| 黄芪秸秆 Astragalus straw | 12.70 |  |
| 玉米 corn | 14.90 |  |
| 麸皮 bran | 7.20 |  |
| 菜籽饼 rapeseed cake | 6.80 |  |
| 豆蛋白粉 bean protein powder | 3.60 |  |
| 食盐 salt | 0.60 |  |
| 预混料 gunk | 1.90 |  |
| 合计 Total/ | 100 |  |
| 营养水平 Nutrient level |  |  |
| 综合净能*（MJ/kg） | 81.54 |  |
| 干物质Dry matter(DM) | 92.97 |  |
| 粗蛋白质Crude protein(CP) | 13.43 |  |
| 粗脂肪Ether extract(EE) | 8.95 |  |
| 中性洗涤纤维Neutral detergent fiber(NDF) | 43.42 |  |
| 酸性洗涤纤维 Acid detergent fiber(ADF) | 21.36 |  |
| 钙Ca | 0.52 |  |
| 磷 P | 0.34 |  |
